# Structure-Bioactivity Relationship of the Functionalized Polysulfone with Triethylphosphonium Pendant Groups: Perspective for Biomedical Applications

**DOI:** 10.3390/polym15040877

**Published:** 2023-02-10

**Authors:** Adina Maria Dobos, Adriana Popa, Cristina Mihaela Rimbu, Anca Filimon

**Affiliations:** 1 Department of Polycondensation and Thermally Stable Polymers, “Petru Poni” Institute of Macromolecular Chemistry, 700487 Iasi, Romania; 2 “Coriolan Dragulescu” Institute of Chemistry, Mihai Viteazul Blv., 24, 300223 Timisoara, Romania; 3 Department of Public Health, University of Life Science Iasi, 8 Mihail Sadoveanu Alley, 707027 Iasi, Romania

**Keywords:** functionalized polysulfones, rheological properties, structural characteristics, morphological aspects, antimicrobial activity

## Abstract

Development of new biomaterials based on polysulfones tailored to act in various biomedical fields represents a promising strategy which provides an opportunity for enhancing the diagnosis, prevention, and treatment of specific illnesses. To meet these requirements, structural modification of the polysulfones is essential. In this context, for design of new materials with long-term stability, enhanced workability, compatibility with biological materials and good antimicrobial activity, the functionalization of chloromethylated polysulfones with triethylphosphonium pendant groups (PSFEtP+) was adopted. The surface chemistry analysis (Fourier transform infrared spectroscopy (FTIR), Energy-dispersive X-ray spectroscopy (EDX)), rheological properties, morphological aspects (Scanning electron microscopy (SEM), polarized light microscopy (POM)), and antimicrobial activity of the synthetized polysulfone were investigated to establish the relationship between its structure and properties, as an important indicator for targeted applications. Based on the obtained features, evaluated by the relationship between the rheological properties and microstructural aspects, and also the response at the biomaterial-bacteria interface, these qualities have been confirmed in their performance, in terms of thermal stability, antimicrobial activity, and also an increase in lifetime. Consequently, derived results constitute the preliminary basis for future tests concerning their functionality as gel matrices in biomedical devices.

## 1. Introduction

Currently, research interest has been focused on the development and operation of analytical devices, including biosensors, immunosensors, and electrochemical sensors, for the detection of important analytes (biological and chemical pathogens or contaminants) [1,2,3,4,5]. These devices are of great use in medicine for medical diagnosis, but also in environmental monitoring and food analysis [6,7,8,9,10,11] due to their sensitivity, specificity, and capacity in real time. The component part consists of a receptor layer and a transducer. The receptor is represented by a biorecognition material that detects the desired analytes, while the transducer transforms the biochemical signal into a digital electronic signal [12]. These qualities make the sensor a perfect tool for diagnosis, prevention, and treatment of specific illness, including infections and autoimmune diseases. Often, biosensors are presented in the form of micro-total analysis systems (μTAS) or lab-on-a-chip (LOC). For the latter, a wide range of materials such as glass, plastic, paper, hydrogels, polymers, or composites can be used [13]. Among these, polymeric materials have received special attention due to their low cost, flexibility, and biocompatibility, presenting advantages in terms of structural stability, dispersion in water, ease of processing, and integration into a detection device [14]. Conductive polymers and those that have the ability to be physically or chemically modified by adding functional groups or biologically active molecules are also of particular importance.

Polysulfones, PSFs, can be mentioned from this class of compounds. They are thermoplastic compounds that, due to special characteristics such as high strength, good electrical characteristics, transparency, chemical and thermal stability provided by aryl groups linked by sulfonyl and ether groups, make them suitable for demanding applications that other polymeric materials cannot satisfy [15,16]. Moreover, due to the chain’s flexibility assured by the ether and isopropylidene groups, PSFs can be processed in different forms: films, fibers or gels. In this context, PSFs, attractive immune-material carriers act not only as a membrane but also as a reservoir for immunological materials (enzymes and antibodies) [17]. Because of its compatibility with a large number of ionophores and organic and biological compounds, polysulfones have been used in humidity sensors, gas sensors, and biosensors [18,19,20]. The sensors will provide information on chemical species (in the liquid or gaseous phase) from a physical, chemical, or biological environment in the form of an electrical signal. 

Obtaining devices designed to meet these requirements involves the appropriate selection of the biomaterials for successful practical application in the health and medical fields. From this perspective, the structural modification of PSFs is essential. Therefore, depending on the nature of the substituents, the way they are obtained (either through chemical and/or physical modifications), and of the resulting properties (e.g., thermal stability, biocompatibility, and specific surface properties), polysulfone-based materials can be used in various fields. In this context, polysulfonic materials containing ammonium or phosphonium groups have gained attention in various technologies, being applied as potential phase transfer catalysts, antistatic agents, biocides, and moisture sensors. In addition, polyelectrolytes are known to have a wide range of applications in the biomedical field as antimicrobial agents, synthetic enzymes, non-viral gene delivery, or as drug carriers. Thus, a cationic structure with unique properties will be obtained as result of the ammonium and phosphonium group electronegativity [21,22,23]. 

Previous studies [24,25,26,27,28,29,30], which focused on the functionalization of polysulfone with ammonium and phosphonium groups, showed an improvement in antimicrobial activity and surfaces decontamination, but also an increase in the lifetime of the materials. In blood-contact applications, the adsorption of serum protein onto polysulfone membranes induces some life-threatening complications. Thus, functionalized PSFs have proven their utility in medical applications, with modification procedures allowing a compromise between hydrophobicity and hydrophilicity in order to improve the blood compatibility of polysulfone materials [24,31]. Compared to macromolecular compounds containing ammonium groups, those with phosphonium groups present superior properties, such as high thermal stabilities, high conductivities, and lower viscosities. Accordingly, considering the special characteristics of functionalized PSFs, the aim of this study was to obtain, by chemical modification of PSF with phosphonium groups, new polysulfonic compounds that can cover the daily needs in various fields. The use of PSFs in the biomedical field as drug carriers, biosensors, or blood contact devices represents a challenge for researchers. These materials have properties that recommend them for this type of application, including blood compatibility, prevention of protein adsorption, and structural similarities to naturally occurring nucleic and teichoic acids [32]. Therefore, our concern was to find the most suitable functionalization compounds so that the resulting polysulfonic materials acquire new improved properties (e.g., thermal stability, antimicrobial activity) which can be successfully applied in the biomedical field. For this reason, the research of this work is focused on obtaining polysulfones by functionalization with triethylphosphonium pendant groups (PSFEtP^+^) and its physico-chemical and biological characterization. All obtained results represent a basis for development of functionalized polysulfones tailored to act in diagnosis and prevention in various biomedical fields, so as to improve the overall quality of life.

## 2. Materials and Methods

Chloromethylated polysulfone (CMPSF, 8.68% Cl, SD = 1.23 [33]), triethylphosphine (Sigma-Aldrich, Burlington, MA, USA, 99%), 1,4-dioxane (Aldrich, p.a.), N, N-dimethylformamide (Sigma–Aldrich, 99%), and diethyl ether (Chimopar, p.a., Bucharest, Romania) were used without purification. 

Chloromethylated polysulfone (CMPSF) was synthesized using UDEL-1700 polysulfone (Union Carbide) with M_n_ = 39,000 g/mol and M_w_/M_n_ = 1.625, purified by repeated reprecipitations in chloroform, and dried for 24 h in vacuum, at 40 °C, before being used. The synthesis reaction took place at 50 °C, in presence of mixture of commercial paraformaldehyde with an equimolar amount of chlorotrimethylsilane (Me_3_SiCl) (chloromethylating agent) and stannic tetrachlor (SnCl_4_) (catalyst) [33]. Subsequently, the chemical modification of the chloromethylated polysulfone with triethylphosphonium groups (PSFEtP^+^) was performed in dioxane, as solvent, using 18 g of CMPSF and 4 mL (PEt_3_). The mixture was kept under stirring, in nitrogen atmosphere for 15 h at 90 °C. During the reaction, a viscous mass of product was obtained, which was filtered, washed with dioxane, and then with ethyl ether, and finally dried. 

Staphylococcus aureus ATCC 25923 (*S. aureus*), Escherichia coli ATCC 25922 (*E. coli*), Pseudomonas aeruginosa ATCC 9027 (*P. aeruginosa*), Candida albicans ATCC 90028 (C. albicans) were used as test microbial strains.

Determination of the chlorine and phosphorus contents of the synthesized polysulfone was performed according to the protocol presented in detail in previous work [34]. A sample from the obtained polymer was burned in an oxygen atmosphere, the gases being adsorbed in aqueous solutions of hydrogen peroxide (0.15 wt.%). The chlorine content was determined quantitatively by potentiometric titration using an aqueous solution of silver nitrate (AgNO_3_) 0.05 M. The phosphorus content was obtained by adsorption in water of phosphorus pentoxide (P_2_O_5_) from a gas sample resulting from the polymer sample burnt in the oxygen atmosphere. The solution obtained was titrated with an aqueous solution of cerium (III) 0.005 M in the presence of Eryochrome Black T as indicator. Thus, the characteristics of the synthesized polysulfones are presented in Table 1. 

The Fourier transform infrared spectrum (FTIR, KBr pellets) of the functionalized polysulfone with triethylphosphonium groups (PSFEtP^+^) was obtained on a JASCO spectrophotometer in the range 4000–400 cm^−1^. The thermal properties of the final product were characterized through TGA/DTA analysis on a TGA/SDTA 851-LF1100-Mettler apparatus at a heating rate of 10 °C/min in a nitrogen atmosphere and temperature range from 25 to 800 °C. 

**Figure 1:**
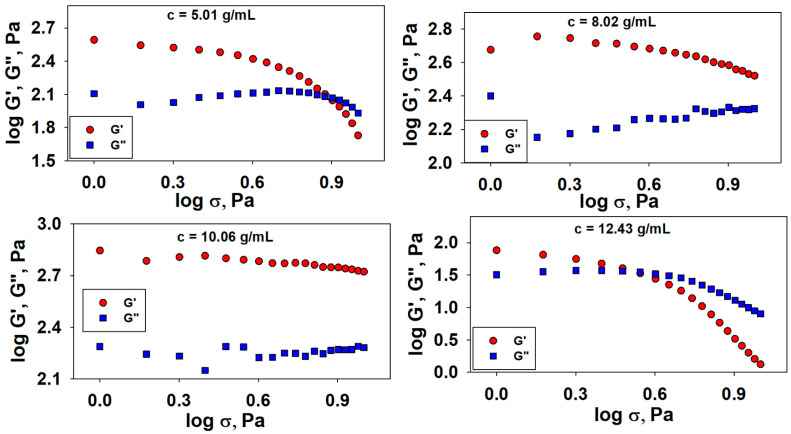
Log–log plots of storage (G′) and loss moduli (G″) versus shear stress (σ) for PSFEtP^+^ solutions at different concentrations.

The rheological behavior, in dynamic regime, of PSFEtP^+^ concentrated solutions obtained in N, N-dimethylformamide (DMF) was analyzed using a Bohlin CS50 rheometer, manufactured by Malvern Instruments (Worcestershire, UK). The measuring system presents cone-plate geometry with a cone angle of 4° and 40 mm diameter. The dynamic viscosity measurements were performed in the range of 0.07–150 s^−1^ shear rate, at different temperatures (25–45 °C) and solution concentrations between 5.01–12.43 g/mL. Also, the oscillatory shear analyses were registered over a frequency range of 0.1–100 Hz, at 25 °C and a shear stress of 2 Pa. The shear stress value was selected as result of a suitable strain amplitude test performed at a frequency of 1 Hz from the linear viscoelastic regime. In this sense, prior to the measurements, a suitable strain amplitude test was performed to find the domain of shear stress in which the storage and loss moduli are constant (Figure 1). 

The supramolecular architecture of the PSFEtP^+^ gels was assessed by polarized light microscopy (POM) on PSFEtP^+^ samples placed between two lamellae, using an Olympus BH-2 polarized light microscope (Olympus BH-2 (Olympus Company) Japan, Tokyo).

The morphology of the PSFEtP^+^ lyophilized gels was investigated using a field emission Scanning Electron Microscope (SEM, EDAX Quanta 200 (FEI Company, U.S., Hillsboro, OR, USA) at an accelerated electron energy of 20 keV. 

**Figure 2:**
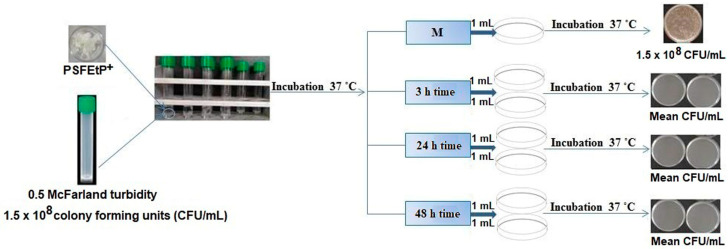
Work diagram: Stages of the experimental protocol.

The antimicrobial potential of the studied polysulfone (PSFEtP^+^) was determined against four microbial strains: *Staphylococcus aureus*, *Escherichia coli*, *Pseudomonas aeruginosa*, and *Candida albicans.* For the experimental tests, 24 h bacterial strains were used, from which microbial suspensions were made in physiological serum. Each bacterial strain was brought to a density of 1.5 × 10^8^ CFU/mL, equivalent to 0.5 McFarland Turbidity Standard. Four types of polysulfones of different concentrations, namely C_1_ = 5.01, C_2_ = 8.02, C_3_ = 10.06, C_4_ = 12.43 g/mL, having the same weight (0.07 g), were made. Samples C_1_–C_4_ were distributed in test tubes with 0.5 McFarland microbial suspension, after which they were incubated (37 °C) for different times (3, 24, 48 h) under aerobic conditions (Figure 2). At the set times, 1 mL from the test tubes, in which there is polysulfone suspended in the microbial suspensions, was taken and distributed in sterile Petri plates, on top of which was added culture medium Mueller Hinton Agar (standardized bacteriological growth medium [35], Oxoid) melted and cooled at 45 °C, so that the microbial cells were not destroyed. After solidification, the Petri plates were incubated at 37 °C for 24 h, after which the microbial load was examined and assessed by counting the colony-forming unit (CFU/mL, standard quantification method used to determine the number of viable cells [36]). The control is represented by the Petri dish in which Mueller-Hinton agar and 1 mL of microbial suspension (1.5 × 10^8^ CFU) were distributed. Since it was worked in duplicate, an average of the values obtained for each time (3, 24, 48 h) and each concentration of polysulfone (C_1_–C_4_) taken into the study was made.

## 3. Results and Discussion

### 3.1. Synthesis and Characterization of Functionalized Polysulfone with Triethylphosphonium Pendant Groups

Polysulfones are known as high-performance materials with specific properties that recommend them to many applications, such as coatings, biomaterials, microelectronic devices, biosensors, filtration membranes, and thin film technology [37,38,39,40,41,42]. Introduction of functional groups onto the polysulfone backbone solves some limitations, and also extends the potential application ranges of these high-performance materials through the specific properties gained. Thus, chemical modifications can be easily achieved via reacting simple chemicals with a reactive pendant group, such as halomethyl, vinyl, and carbonyl present on the polymer. Among these reactive groups, the chloromethyl group is especially useful. The advantage of polysulfone modification by chloromethylation reaction is represented by the property of chloromethyl groups, which can be easily substituted with different nucleophilic agents [43,44]. Thus, a potential strategy aimed at synthesizing/developing new applicable polysulfone materials in various fields/technologies consists in the functionalization of the intermediary polysulfone containing chloromethyl groups with various monomers, which will yield improvements of the chemical, thermal, and biological properties. Therefore, chloromethylated polysulfones are important both by the fact that they represent the path to a great structural diversity, but also through the additional benefits of the obtained products which allow increased applicability in various fields.

**Scheme 1:**
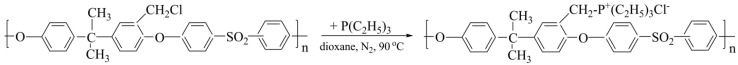
Reaction scheme for obtaining functionalized polysulfone with triethylphosphonium pendant groups.

In this sense, having as the main objective the exploration of the potential use of the functionalized polysulfone materials for biomedical applications, we chemically modified chloromethylated polysulfone (CMPSF) with triethylphosphonium groups (Scheme 1).

**Figure 3:**
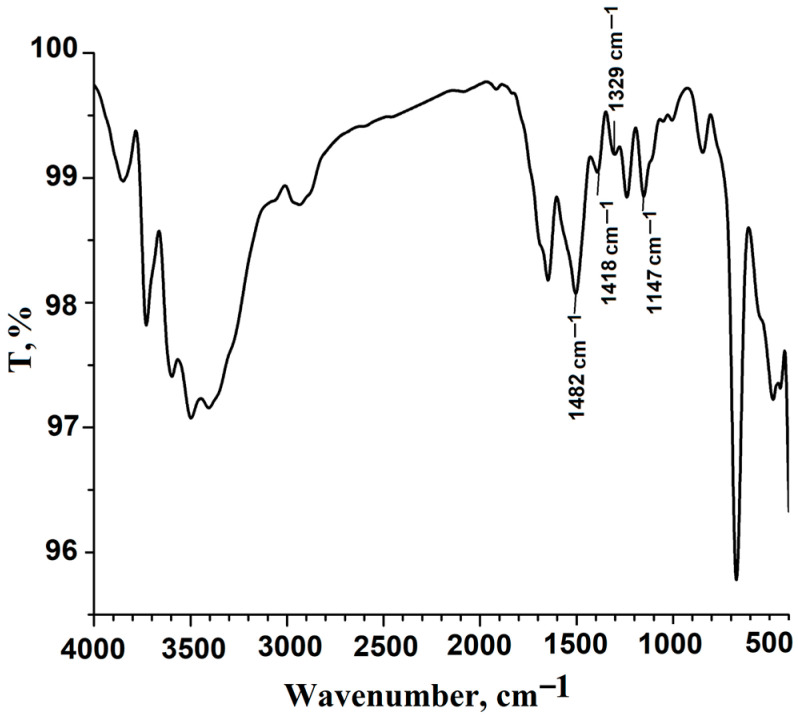
FTIR spectrum of polysulfone functionalized with triethylphosphonium groups.

**Figure 4:**
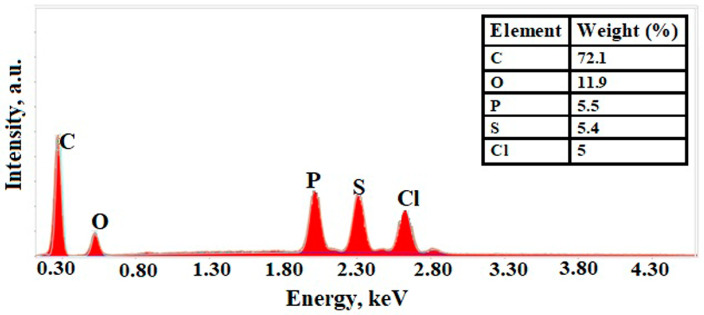
EDX spectra for polysulfone functionalized with triethylphosphonium groups.

An accurate characterization of the chemical structure of the functionalized polysulfone was performed by FTIR and EDX methods (Figure 3 and Figure 4). According to Figure 3, the FTIR spectrum presents the absorption bands around 3200–3600 cm^−1^ for alkyl groups (–CH_2_, –CH_3_) from the ethyl radical. The aromatic structure is confirmed by the appearance of the absorption band at approximately 1582 cm^−1^ and the absorption band characteristic for −SO_2_ asymmetric stretching appearing at 1238 cm^−1^. The reference peaks from the FTIR spectrum of PSFEtP^+^ show absorption bands around 1482 and 1329 cm^−1^ assigned to the methyl asymmetric and symmetric (–CH_3_) groups, respectively, and 1418 cm^−1^ corresponding to the methylene bending (–CH_2_–) from the triethylphosphonium group. Additionally, the absorption bands characteristic of −P-CH_2_ appear at 1147 cm^−1^, and for out-of-plane −CH of benzene at 843 and 688 cm^−1^. Thus, spectroscopy measurements have confirmed the formation of phosphonium groups.

Additional information which confirms the occurrence of a new functionalized polysulfone by the reaction of the chloromethylated polysulfone (CMPSF) with triethylphosphonium groups, according to Scheme 1, was obtained by EDX analysis (Figure 4). Thus, by analysis of the chemical structure of functionalized polysulfone with triethylphosphonium groups, all elements identified in EDX spectra (Figure 4) confirm the functionalization by the presence and content of phosphorus from triethylphosphonium groups.

**Figure 5:**
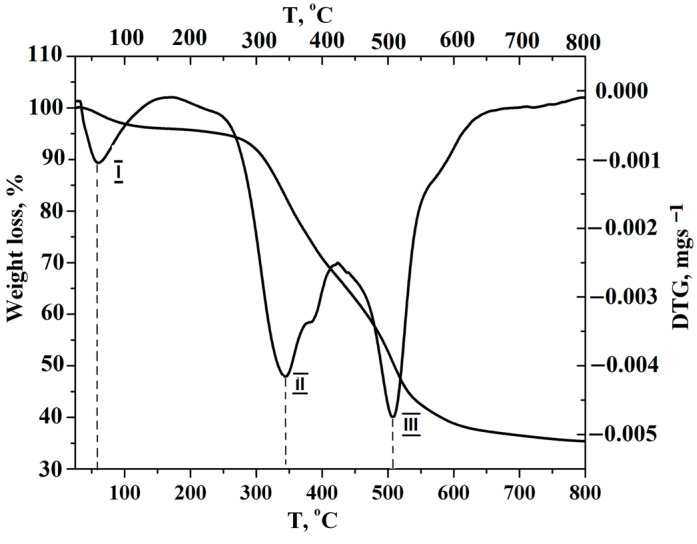
TG-DTG curves for polysulfone functionalized with triethylphosphonium groups.

Compared to the chloromethylation reaction, this functionalization path represents another level of structural modeling with an impact on the properties, by obtaining new functional polymeric materials. The strategy of synthesis of this new class of ionic polymers allows the improvement of thermal stability, being preferred for long-term use applications [26], for facilitating aggregation [27], or as an aid in matrix reinforcement of ionomers [28]. Thus, through this chemical modification, the side group contributed to the skeleton rigidity which is why the phosphonium-functionalized polysulfone exhibits improved thermal, mechanical, and electrochemical stability [45]. In the present study, the thermogravimetric analysis allowed the highlighting of the thermal decomposition stages (Figure 5). According to the thermogram, the thermal degradation occurs in three steps with various weight losses, as can be seen from the data presented in Table 2. Thus, when a sample is heated, at a temperature of about 58 °C, a peak corresponding to the solvent and water removal appears. As the temperature increases (around 400 °C), new peaks, which can be attributed to the functional group elimination, were visualized. Continuing the sample heating up to 600 °C, a peak associated with breaking of the polysulfone main chain was indicated.

Additionally, according to the data from Table 2, it can be stated that compared to PSFQ [46] (Figures S1 and S2, Supplementary Materials), phosphonium-functionalized polysulfone exhibits a higher glass transition temperature. This aspect allows us to support the hypothesis, according to which the polymers carrying these groups have different properties from those of other functionalized polysulfones, which is why the interest in the investigation of alternative phosphonic “protogenic” groups has increased [47,48].

### 3.2. Conformational Characteristics of Functionalized Polysulfone with Triethylphosphonium Pendant Groups Evaluated by Rheological Parameters

#### 3.2.1. Dynamic Viscosity

**Figure 6:**
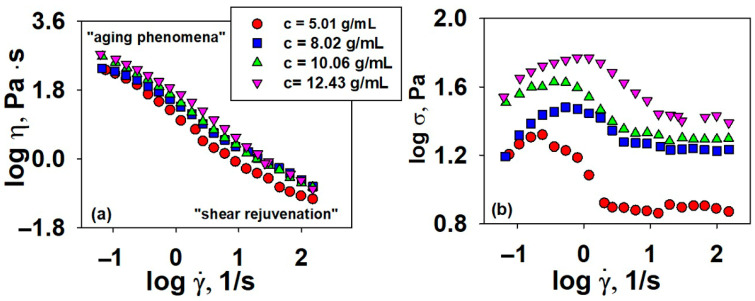
Log–log plots of dynamic viscosity, η, (**a**) and shear stress, σ, (**b**) function on shear rate,$\;\dot{\gamma }$, for PSFEtP^+^ at 25 °C in DMF at different concentrations.

Generally, the conformational characteristics of macromolecular chains in solutions, under shear conditions, are dictated by the interactions of different types of molecules, and have an important impact in defining the properties of the final materials with applicability in different fields. In this context, the rheological response, in terms of dynamic viscosity and oscillatory shear parameters of polysulfone containing triethylphosphonium pendant groups in DMF, is important to know. Based on these considerations, the flow curves from Figure 6 show the variation of the dynamic viscosity and of shear stress vs. shear rate for PSFEtP^+^ solutions of different concentration, in DMF, at 25 °C.

Examination of dependences from Figure 6 reveals that studied solutions exhibit a shear thinning behavior, with the dynamic viscosity decreasing gradually with increasing shear rate. The high values of the dynamic viscosity in the field of low shear rates show the need for applying a high shear stress to destroy the strong network between the polymer particles, and to orient them in the flow direction. Thus, because of the microstructure solution destruction as the shear rate increases, the dynamic viscosity decreases in the samples, indicating a pseudoplastic behavior. 

Variation of the dynamic viscosity is difficult to predict in the current system; an increasing concentration results in multiple interactions, which has an effect on the rheological behavior. In the literature, there are several structural models based on qualitative concepts, which refer to the impact of the interactions of the analyzed polymeric systems on the mechanical behavior [49,50], but a phenomenological understanding of this behavior is followed. Thus, for the studied polysulfone solutions, it is observed that for high concentrations, namely concentrations that exceed 10 g/mL (i.e., 10.06 g/mL and 12.43 g/mL), the polymer chains can interact with each other even in the absence of a shear rate action. In this case, the viscosity of the solutions depends very much on the strength of the interactions established in the system, which extends throughout the sample as the concentration increases. In the absence of any stress, the polymeric systems will tend to a state of equilibrium as a result of local and collective rearrangements. For less concentrated solutions which do not have a compact network, and that are subjected to shear rate, they will continue to flow, although in this state there may be significant internal stresses generated by the spatial distribution of the polymer chains. The application of a shear rate has the effect of weakening the interactions that are established between the polymeric chains, and thus to a destabilization of the compact gel network [51,52]. Based on these statements, we can assume that the flow behavior of PSFEtP^+^, in terms of dynamic viscosity, results from the competition of two opposite processes: “aging phenomena”—characterized by high viscosity values due to the formation of a microstructure, and “shear rejuvenation”—characterized by a decrease in viscosity in time under shear as result of microstructure solutions destruction (Figure 6a).

In addition, it can be observed that the dynamic viscosity increases with increasing concentration. This can be explained by the fact that the increased number of voluminous triethylphosphonium pendant groups leads to a decrease of free volume, and an intensification of the intra- and intermolecular interactions. Thus, besides the bonds established between the polymer/solvent, new strong intramolecular interactions of electrostatic nature occur between the phosphonium groups, which will decrease the flexibility of the polymeric chains leading to an increase in dynamic viscosity. The studied polysulfone represents a pseudoplastic material characterized by an entanglement density, and an enhancing number of oriented segments as a result of the shear rates increasing [53,54]. The higher orientation of the polymer chains is the major cause of the non-Newtonian behavior. 

On the other hand, in the description of the rheological behavior of some fluids under the shear rate action, different models such as Herschel-Bulkley, Ostwald, Carreau-Yasuda, Bingham model, Kundu and Cohen and Tropea et al., can be used [55,56,57]. In this context, flow behavior illustrated in Figure 6a is also reflected in the values obtained for the flow behavior (n) and consistency (k) indices, evaluated by applying the Ostwald-de Waele model (Equation (1) on experimental data corresponding to the linear dependence from double logarithmic plot of shear stress versus shear rate (Figure 6b).

(1) $$\sigma =k\cdot {\;\dot{\gamma }}^{n}$$

Thus, the obtained data for the studied polysulfonic systems indicate flow index values varying between 0.22 and 0.31, which means that the resistance of the solutions to the shear rate action decreases, this being correlated with a pseudoplastic character. Moreover, the high values of the consistency index (varying between 29.51 and 83.17), are explained by enriching the studied solutions in phosphonium groups, as the concentration increases. These groups will generate additional interactions and entanglements leading to the appearance of a compact gel structure. 

**Figure 7:**
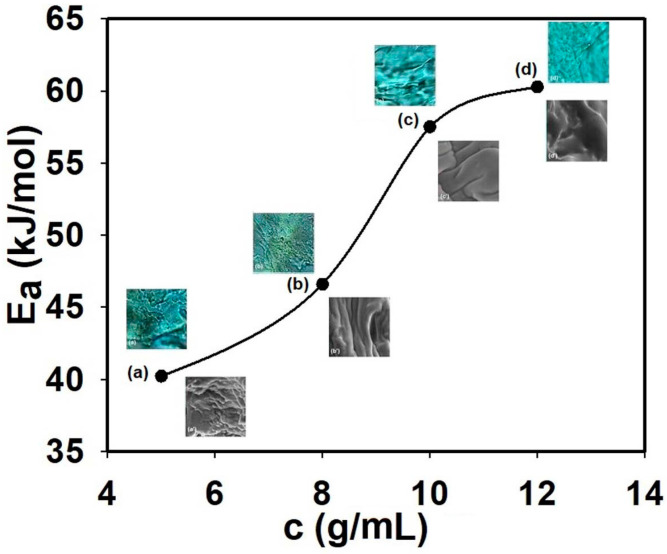
Dependence of activation energy as a function of PSFEtP^+^ solution concentration in DMF at 25 °C. Small images represent POM and SEM images recorded for PFEtP^+^ samples at studied concentrations: 5.01 g/mL (a), 8.02 g/mL (b), 10.06 g/mL (c), 12.43 g/mL (d).

The intensification of interactions from the system established between the chain segments, with an increase of the solution concentration, can be also described by an apparent activation energy, E_a_, (Figure 7) that is given by the Arrhenius equation (Equation (2)), which represents the energy necessary for the movement of an element of the fluid [58,59]:

(2) $$\ln\eta ={\ln\eta }_{0}+E_{a}/\mathrm{RT},$$

where: η_0_ represents a pre-exponential constant, R is the universal gas constant, and T is the absolute temperature. 

**Figure 8:**
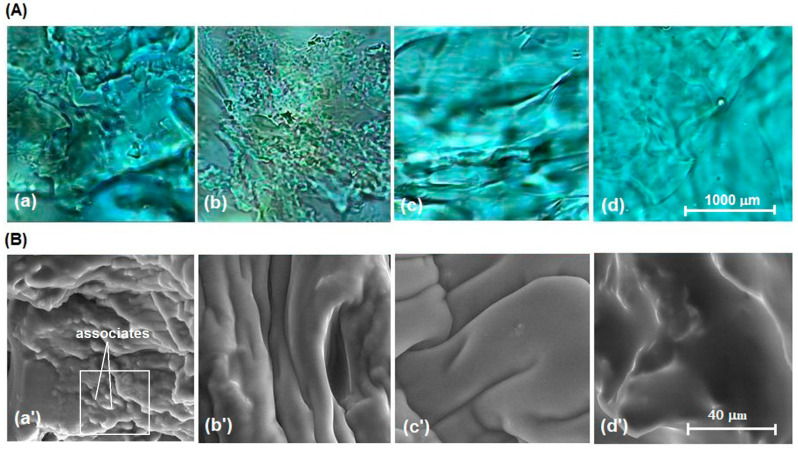
POM (**A**) and SEM (**B**) images for PSFEtP^+^ solutions and corresponding lyophilized samples obtained in DMF at different concentrations: 5.01 g/mL (**a**,**a’**), 8.02 g/mL (**b**,**b’**), 10.06 g/mL (**c**,**c’**), 12.43 g/mL (**d**,**d’**).

Increasing of the activation energy as the solutions concentrate, and implicitly the number of phosphonium groups increase, emphasizes the appearance of some steric hindrances generated by the voluminous groups, but also the intensification of the interactions from the system. Also, proof of the intensification of the interactions in the polysulfonic systems as the concentration increases, responsible for the establishment of a gel network [60], has been confirmed by comparative investigations using the polarized light microscopy and scanning electron microscopy analysis (Figure 8). 

As the literature mentions, a physical gel may appear either as result of the phase separation process, or due to the increase in polymer solution concentrations [61,62]. Thus, as can be visualized from the recorded images for PSFEtP^+^ solutions at low concentrations (Figure 8A(a,b),B(a’,b’)), non-uniformities are observable, while increasing the concentration (Figure 8A(c,d),B(c’,d’)), fine textures with bands characteristic to layered architectures appear. The latter morphologies indicate the presence of anisotropic assemblies in high concentration solutions, which come from polymer/solvent, polymer/polymer bonding networks.

Therefore, SEM image characteristics for less concentrated solutions (Figure 8A(a’)) show the presence of associates generated by the system interactions, and also the appearance of some narrow bands (Figure 8B(b’)) which highlights that the gel structures begin to form even from low concentrations. This is explained by the fact that, in the studied systems, the presence of voluminous phosphonium groups can generate rigid conformations by intensifying the intramolecular, but also the intermolecular, interactions process that is more evident with increasing concentration. Thus, it can be assumed that the associates (Figure 8A(a’)) and, implicitly, the strong electrostatic interactions established between the polysulfone chains containing phosphonium groups, which intensifies with increasing concentration, determine the transition from sol to gel. In this context, as images show for high concentrations (Figure 8B(c’,d’)), the bands begin to enlarge, suggesting the formation of a more compact gel structure. In addition, as a consequence the associates generated by intramolecular interactions tend to be incorporated into the dense structure of the gel, so that in microscopy images (Figure 8B(b’–d’)) they become less visible.

Consequently, the images obtained by both types of optical measurements support the conclusions of the rheological tests, according to which a competition between the two processes generated by the chemical and physical responses of polymer to cumulative effects of the specific interactions exist, and as the concentration increases this led to gel structure formation. 

#### 3.2.2. Oscillatory Shear Parameters

Viscoelastic measurements can significantly contribute to the knowledge and differentiation of polymer systems, completing the rheological studies developed in shear regime. The evaluation of the oscillatory shear parameters provides information concerning modification of solution properties, which include the transition from viscoelastic to gel behavior, as the PSFEtP^+^ solution concentration increases. Usually, polysulfone solutions are Newtonian liquids, but depending on the nature of the substituents they may exhibit an elastic behavior. For example, in previous rheological studies performed on chloromethylated polysulfone, this behavior was not observed [63], while for polysulfones containing phosphorus pendant groups [60] elastic properties were identified.

**Figure 9:**
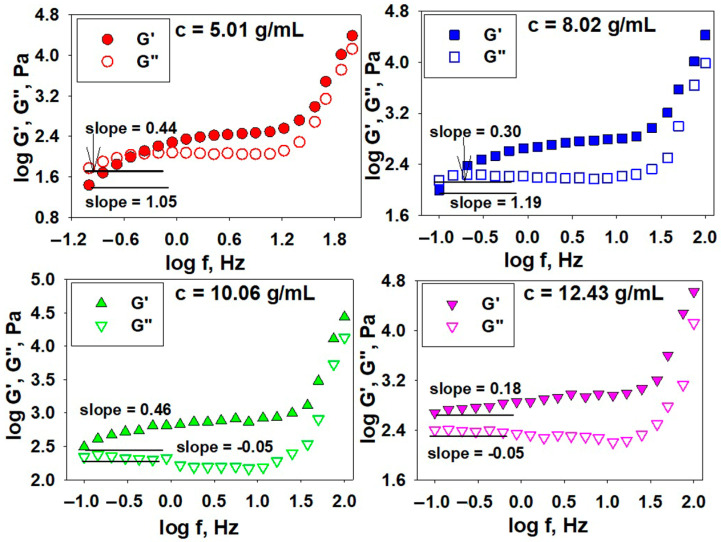
Log–log plots of storage (G′) and loss moduli (G″) *versus* frequency (f) for PSFEtP^+^ solutions at different concentrations.

In the present study, the oscillatory tests were performed in order to obtain information on the structural changes induced by the molecular interactions, and to analyze the crosslinking behavior of the polysulfone solutions. Figure 9 represents the dependence of the dynamic moduli (storage (G′) and loss (G″) moduli) as a function of frequency. This dependency provides information about the nature of the system and can predict whether it is a dilute solution, a tangled network, a weak gel, or a strong gel [64,65]. According to the literature, for less concentrated solutions, G″ > G′ over the entire frequency range, for the entanglement network the two moduli intersect at a point belonging to this frequency domain, confirming the tendency of more solid-like behavior at higher frequencies [66]. On the other hand, when G′ > G″ and approximately parallel to each other, the systems present characteristics of gels. The difference between a weak gel and strong gel consists in the fact that the G′ slope is zero, while G″ shows a minimum for low frequency values [67].

As can be seen from our data (Figure 9), both moduli are dependent on frequency, increasing with it. Also, the influence of concentration on the moduli is significant. For low concentrations, in low frequency domains, the storage moduli (G′—that represents the stored energy and recovered deformation as elastic behavior) is lower than loss moduli (G″—that describes the dissipated energy and irreversible deformation) which means that a viscous response predominated. The elastic response prevails at high frequency, so that at an intermediate value a crossover in the two moduli exists [68]. This viscoelastic behavior is a characteristic of an entangled system and can be explained as follows. At relatively low frequencies the unentangled polysulfonic chains can easily exist during the period of oscillation, the viscous storage being dominant at the imposed deformation. As the frequency increases, the entanglement becomes a temporary association of chains and generates a three-dimensional network in which the storage moduli prevail [69]. Increasing concentration leads to G′ > G″ over the entire frequency range, suggesting a gel characteristic, where in the linear domain the deformation will be essentially elastic or recoverable. This rheological behavior has been attributed mainly to phosphonium groups belonging to polysulfone, and shows that a temporary network can be improved by increasing the concentration, with sol-gel transition taking place between 8.02 and 10.43 g/mL.

In addition to those mentioned, the dynamic moduli present a power law dependence on frequency as $G^{\prime }≅G^{\prime \prime }\sim f^{x}$. The x exponent is given by the slope values of G′ and G″ curves and provides information on the microstructural changes that take place in the systems, as the solution concentration increases. As can be seen, at low concentrations this parameter is greater for G′ than for G″, highlighting the characteristics of a viscoelastic fluid. Decreasing of the x values, along with higher values of G′ than that of G″, over the entire frequency range, is the response to gel behavior resulted from the interactions among the molecules and entanglements. Slightly higher values of G′ compared to those of G″ show an increase in crosslinking density, and implicitly of elastic characteristics, the viscous behavior starting to disappear [70]. The nonlinear evolution of the G″ in low frequency domain, starting from low concentrations of polysulfone solutions, suggests the beginning of the structural changes in the system, equivalent to the transition from a viscous to elastic state. 

As a consequence, it is observed that the frequencies corresponding to the crossover point, which delimits the viscous flow from elastic, and for which G′ = G″ moves to lower frequency values as the concentration increases, so that for high concentrations it disappears. In other words, a small increase of concentration shows only a slight tendency towards a gel network, ensured by the bonds established between the polymeric chain and solvent. At the same time, the voluminous nature of the phosphonium groups may represent a reason for this behavior. On the other hand, increasing solution concentration led to an increase of the number of phosphonium pendant groups and consequently, to intensification of interactions from the system and macromolecular rearrangements, which will generate a more compact network with gel characteristics. Thus, for high concentrations, the specific structural changes are determined both by the interactions established between the polysulfonic chains and the solvent molecules, but also by those established between the polysulfonic chains themselves. The high values of G′ compared with G″ for the highest concentration may represent a proof of irreversible deformation of polysulfone solutions [71]. Thus, the resistance to deformation and flow of the self-crosslinking polysulfone solutions had been enhanced with increasing concentration. Therefore, it can be assumed that the studied solutions are divided into two types of systems, namely: a weakly linked system and a strongly linked system. The polysulfone solutions of low concentration (c = 5.01 and 8.02 g/mL) are poorly linked systems, characterized by a poorly structured network, in which predominate weak physical interactions such as London and van der Waals. These interactions are established mainly between the polysulfone chain backbones and the solvent. Compared with these, the polysulfone solutions of high concentration (c = 10.06 and 12.43 g/mL), as a result of the increased number of phosphonium pendant groups, are characterized by new interactions of electrostatic nature, established between the polysulfone chains which imprint on the system a strong structured network. On the chosen frequency range, this behavior can be characterized by a power law relationship (Equation (3)) between the dynamic complex modulus G* and the oscillation frequency, f [72,73,74].

(3) G*=[G′(f)]2+[G″(f)]2=Af1z

where: A—represents a measure of the strength of the interactions from the systems, z is the network extension and is correlated with the number of units from the systems that interact. 

**Figure 10:**
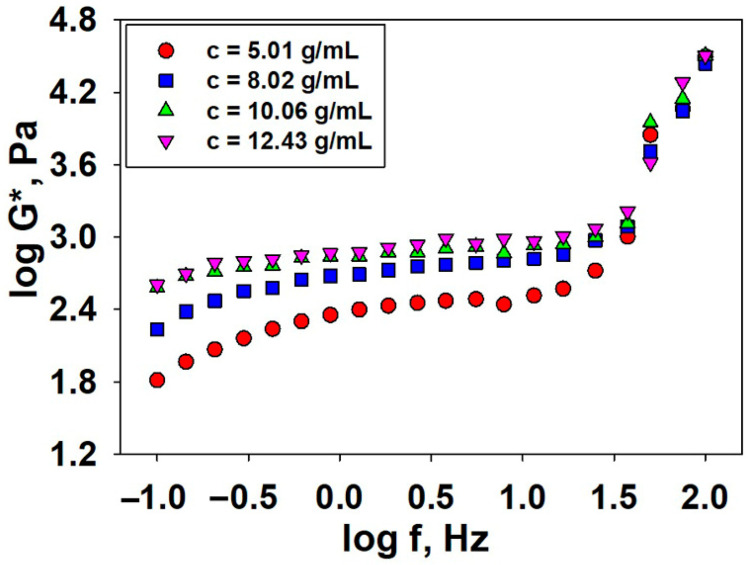
Log–log plots of complex moduli vs. frequency for PSFEtP^+^ solutions at different concentrations.

Thus, by representing the dynamic complex module (G*) as a function of frequency (f), (Figure 10), A and 1/z parameters can be obtained, data being tabulated in Table 3. According to the obtained results, the values of A parameter increase as the PSFEtP^+^ solution concentration increases, this suggesting an intensification of the interaction forces within the network. Moreover, high values of z for the concentrated solution suggest a broad network extension. Therefore, at the molecular level, the gel behavior is correlated with the intense intermolecular interactions and interpenetrations between the polysulfonic chains.

**Figure 11:**
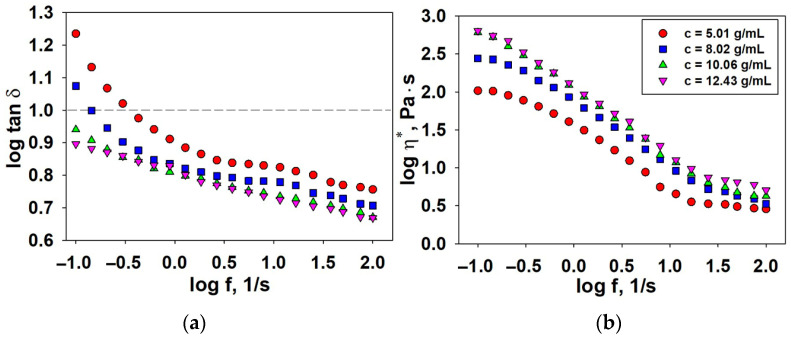
Log–log plots of loss tangent (tan δ, (**a**)) and complex viscosity (η*, (**b**)) vs. frequency for PSFEtP^+^ solution at different concentrations.

In addition to those mentioned, a quantitative evaluation of the viscoelasticity can also be performed using relative magnitudes of G′ and G″ represented by loss tangent (tan δ = G″⁄G′). Figure 11 shows the variation of this parameter and of complex viscosity function on frequency. According to the literature, in low frequency domain, values of tan δ > 1 evidence a viscous behavior, while for tan δ < 1 the systems have characteristics specific to gel [75]. 

As can be seen from Figure 11a, for polysulfone solutions of low concentrations, in low frequency domain, tanδ takes values higher than one, which means that a viscoelastic behavior persists. Increasing of solution concentration leads to values of tanδ lower than one, the shape of the curves became almost horizontal indicating that the crosslinked polysulfone solutions tended to exhibit gel behaviors. From studies performed, it was observed that both the x exponent, resulted from $G^{\prime }\sim f^{x}$, as well as tanδ have decreased as the concentration of polysulfone solutions have increased from 5.01 to 12.43 g/mL. This confirms that an increase of the phosphonium pendant groups and, implicitly, intensification of intra- and intermolecular interactions from the system lead to self-crosslinking solutions, effectively contributing to the expansion of gel networks. These conclusions are also in agreement with the results obtained from the evaluation of the complex viscosity as a function of frequency (Figure 11b). The higher values of η* obtained for the concentrated polysulfone solutions highlight the higher resistance to flow, due to the slowing of the irreversible processes of energy dissipation. As a result, the movement of macromolecular chains generate, in turn, intense intermolecular interactions and macromolecular entanglements [75]. Therefore, the decrease in complex viscosity with increasing shear rate, and its increase with increasing concentration, also represents a feature of pseudoplastic behavior. 

Consequently, the rheological behavior evaluation is of particular interest, especially in the processing and establishment of polymeric materials properties. Thus, the obtained results by the combination of bulk and surface properties of functionalized polysulfone with triethylphosphonium pendant groups represent the basis for future tests regarding successful application in a biological environment. 

### 3.3. Testing the Antimicrobial Capacity

Infections caused by pathogenic microorganisms are of a great concern in many fields, particularly for medical devices, surgical equipment, healthcare products, hygienic applications, water purification systems, textiles, food packaging and storage, major or domestic appliances, and aeronautics [76,77]. Thus, the use of potent and/or specific antimicrobial systems will help to mitigate, combat, and/or eradicate these infections, which means an improvement in the state of well-being. 

**Figure 12:**
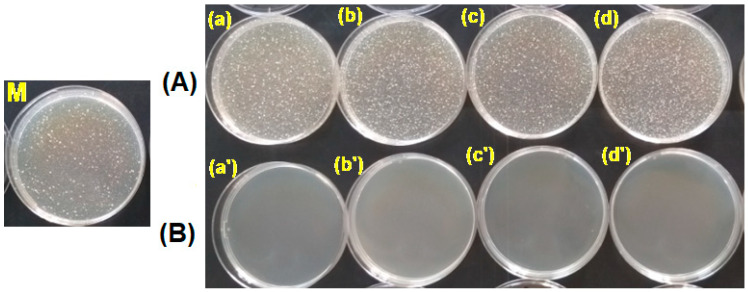
Antimicrobial activity of PSFEtP^+^ polysulfone at different concentrations: C_1_ (**a**,**a’**), C_2_ (**b**,**b’**), C_3_ (**c**,**c’**), C_4_ (**d**,**d’**) against *Staphylococcus aureus* for different times, (**A**) T_1_ = 3 h and (**B**) T_2_ = 24 h, compared to control sample (M).

**Figure 13:**
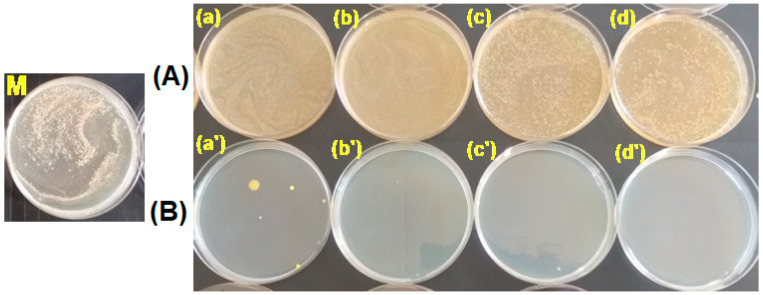
Antimicrobial activity of PSFEtP^+^ polysulfone at different concentrations: C_1_ (**a**,**a’**), C_2_ (**b**,**b’**), C_3_ (**c**,**c’**), C_4_ (**d**,**d’**) against *Escherichia coli* for different times, (**A**) T_1_ = 3 h and (**B**) T_2_ = 24 h, compared to control sample (M).

**Figure 14:**
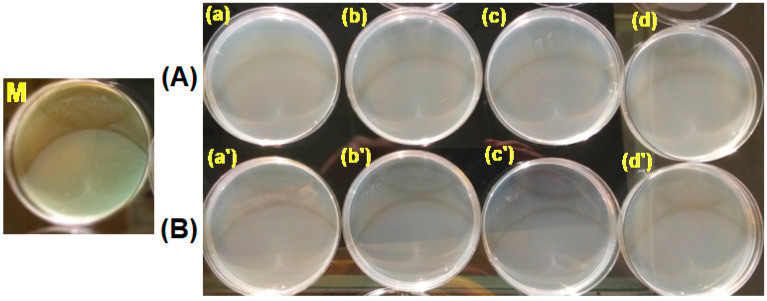
Antimicrobial activity of PSFEtP^+^ polysulfone at different concentrations: C_1_ (**a**,**a’**), C_2_ (**b**,**b’**), C_3_ (**c**,**c’**), C_4_ (**d**,**d’**) against *Pseudomonas aeruginosa* for different times, (**A**) T_1_ = 3 h and (**B**) T_2_ = 24 h, compared to control sample (M).

**Figure 15:**
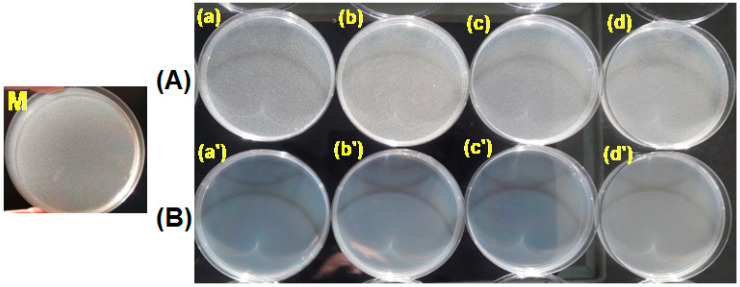
Antimicrobial activity of PSFEtP^+^ polysulfone at different concentrations: C_1_ (**a**,**a’**), C_2_ (**b**,**b’**), C_3_ (**c**,**c’**), C_4_ (**d**,**d’**) against *Candida albicans* for different times, (**A**) T_1_ = 3 h and (**B**) T_2_ = 24 h, compared to control sample (M).

Based on previous studies [24,25,46], it is well known that some positively charged polymeric materials present antibacterial properties and, as a result of their charge, can kill bacteria or prevent their attachment [77]. In this context, it is generally accepted that the bactericidal action mechanism of polycationic materials involves destructive interactions with the bacterial cell walls (negatively charged, containing phosphatidylethanolamine as major component) and/or cytoplasmic membranes [78]. In this regard, functionalized polysulfones with quaternary ammonium groups (PSFQ) have been intensively explored as polymeric biocides, offering some advantages including good antimicrobial activity, films and fibers forming capacity, and favorable hydrophilicity [25,46]. Nevertheless, despite their numerous positive properties, the antimicrobial activity of the polymers with quaternary ammonium salts is not efficient against a wide range of bacteria compared to other polymeric compounds. Quaternized polysulfones exhibit less intense antimicrobial effects generated by the quaternary nitrogen, which is pendant, away from the backbone chain. Therefore, for an enhanced biocidal effect, another molecularly designed antimicrobial polysulfonic system has been established, containing non-soluble quaternary phosphonium salts grafted on the backbone chain, namely, functionalized polysulfone with triethylphosphonium pendant groups (PSFEtP^+^). Under these conditions, the structure-bioactivity relationship of synthetized polysulfone with triethylphosphonium pendant groups was investigated against *Staphylococcus aureus*, *Escherichia coli*, *Pseudomonas aeruginosa*, and *Candida albicans* bacterial strains. As is shown in Table 4 and Figure 12, Figure 13, Figure 14 and Figure 15, a very good antimicrobial activity of PSFEtP^+^ against all tested microbial species has been evidenced. 

The analysis of the obtained data indicates that, after 24 h of contact with the bacterial strains, different concentrations of polysulfones have the ability to stop the multiplication of *Staphylococcus aureus*, *Pseudomonas aeruginosa*, and *Candida albicans*. The exception is *Escherichia coli*, against which the antimicrobial effect was different depending on the polysulfone concentrations and contact time. Thus, after 24 h, for a PSFEtP^+^ sample at concentration C_1_ = 5.01 g/mL, the microbial load was reduced from 1.5 × 10^8^ CFU/mL to 5 × 10^0^ CFU/mL microbial suspension. Instead, for PSFEtP^+^ sample at concentrations C_2_ = 8.02 g/mL and C_3_ = 10.06 g/mL, the viable microbial load was 1 × 10^0^ CFU/mL. Polysulfone at a concentration of 12.43 g/mL was found to inhibit 100 % of the development and multiplication of *Escherichia coli* species.

After 48 h of contact with samples, the antimicrobial effect against all tested microbial strains was maintained. These results highlight the special antimicrobial properties of functionalized polysulfone with triethylphosphonium pendant groups against Gram-positive bacteria (*Staphylococcus aureus*), Gram-negative bacteria (*Escherichia coli*, *Pseudomonas aeruginosa*), and yeasts (*Candida albicans*).

It is known that the cell wall of both Gram-negative and Gram-positive bacteria presents specific compositions, namely the component of Gram-positive bacteria cell walls is peptidoglycan, which confers to them a hydrophobic character, whereas the major constituent of Gram-negative bacteria cell walls is peptidoglycan together with other components such as lipopolysaccharides and proteins, assuring their hydrophilic character [79]. In this context, the different chemical compositions and structures, cell-wall permeabilities, physiologies, metabolisms, and pathogenicities [80] of bacteria generate different antimicrobial activity. This assertion was supported by the hydrophobic nature of the studied polysulfone, the enlargement of the polymeric coil and increase of the charge density, generated by the active cationic centers present on a single polyelectrolyte molecule. Consequently, these polymers exhibit a high biocidal activity as long as the polymeric chain retains positive charge density, whereas some active phosphorous centers unscreened by counterions are also present. On the other hand, cell membranes carry excessive negative charges and the counterions do not interact with membranes under physiological conditions. In a slightly acidic medium, the phospholipids of the cell wall are protonated and the counterions can interact with the membrane. In addition to the electrostatic interactions, these interactions also involve hydrogen bonds and hydrophobic bindings [81]. Therefore, the antimicrobial function of the obtained novel materials can be considered a potential tool in limiting diseases transmission, assuming that they could reduce the population of microorganisms. 

## 4. Conclusions

In this work, new polysulfones with properties required for applications in various fields were synthetized by functionalization of chloromethylated polysulfone with triethylphosphonium groups, and were tested in terms of their rheological properties, morphological aspects, and interactions with different bacterial strains. The effect generated by the phosphonium groups played an extremely important role in the development of bioinspired and sustainable materials, as a result of improved thermal stability, surface characteristics, and biological performance. The quantified results of the obtained parameters by physico-chemical and biological characterization of the PSFEtP^+^ can be summarized as follows:

Analysis of the surface chemistry confirms the successful functionalization of PSFEtP^+^ by the presence and content of phosphorus from triethylphosphonium groups. Thus, the introduction of the functional groups onto a polysulfone backbone solves some limitations, and also extends the potential application range of these high-performance materials through the specific properties gained (long-term stability, improved antimicrobial activity, and also an increase in lifetime).

The rheological response of studied systems, in terms of dynamic viscosity, dynamic modulus, complex dynamic modulus, and complex viscosity, shows the effects of intra- and intermolecular interactions on the structural rearrangements, responsible for the transition from sol to gel as the solution concentration increases. The conclusions of the rheological tests were supported by microscopy analysis. The obtained images show that with the increase of the analyzed polysulfonic solutions concentration, a much more compact structure with finer textures and bands characteristic of layered architectures formed as a result of the strong electrostatic interactions. These parameters have an important impact on processing and defining the properties of the final polysulfonic materials with applicability in different fields.

As an initial attempt to explore the applicative potential of PSFEtP^+^ in the biomedical field, the evaluation of the antibacterial activity using *Staphylococcus aureus*, *Escherichia coli*, *Pseudomonas aeruginosa*, and *Candida albicans* bacterial strains was performed. The obtained results constitute the basis for future tests regarding successful application in a biological environment.

Consequently, it can be said that the main criteria required for the development of polysulfonic biomaterials were fulfilled, as demonstrated by the obtained functionalized model that possesses an attractive combination of properties. The obtained results for functionalized polysulfone with triethylphosphonium pendant groups by the combination of bulk and surface properties represent an important indicator for their targeted application. Thus, the performed studies demonstrate the promising performance and functionality of PSFEtP^+^ to be used in future biomedical applications.

## Figures and Tables

**Table 1:** Characteristics of the synthesized polysulfones: number-average molecular weights (M_n_), total chlorine (Cl), ionic chlorine (Cl_i_) and phosphorus (P) contents, substitution degree (SD), and functionalization degrees (FD) for chloromethylated (CMPSF) and functionalized polysulfone with triethylphosphonium groups (PSFEtP^+^).

Properties	Sample	
	CMPSF	PSFEtP^+^
$\bar{M_{n}}$ (g/mol)	29,000	28,000
Cl (%)	8.68	5.85
Cl_i_ (%)	-	4.23
P (%)	-	3.11
DS (%)	98	72
FD (mmol/g)	-	1.001

**Table 2:** Thermogravimetric data^1^ for polysulfone functionalized with triethylphosphonium groups.

Stage of Thermal Degradation	T_i_ (°C)	T_peak_ (°C)	T_f_ (°C)	Weigh Loss (%)	Weight Loss after Decomposition (%)
I	40	58	160	4.04	64.66
II	160	343	420	28.80	
III	420	505	740	31.82	
		465 *			

T_i_—Temperature at which thermal degradation begins in each stage; T_f_—Temperature at which thermal degradation finishes in each stage, T_peak_—The thermal degradation peak corresponding to the maximum rate of weight loss, the weight loss corresponding to stages of thermal degradation (I, II, III), and the weight loss after decomposition. * According to previous data [46] performed by quaternized polysulfones with ammonium groups (PSFQ).

**Table 3:** Parameters specific to dynamic complex moduli highlighting the sol-gel transition.

Concentration (g/mL)	A	z	Regression
5.0	2.38	2.44	0.984
8.0	2.71	2.94	0.984
10.0	2.86	3.13	0.969
12.0	2.91	3.23	0.975

**Table 4:** Antimicrobial activity of PSFEtP^+^ for different concentrations (C_1_–C_4_) against standardized microbial species.

Bacterial Strains	Time	C1 = 5.01 g/mL	C2 = 8.02 g/mL	C3 = 10.06 g/mL	C4 = 12.43 g/mL
	(h)	(CFU/mL)	(CFU/mL)	(CFU/mL)	(CFU/mL)
*S. aureus*	Control sample	1.5 × 10^8^	1.5 × 10^8^	1.5 × 10^8^	1.5 × 10^8^
	T_1_ (3 h)	≈1.5 × 10^8^	≈1.5 × 10^8^	≈1.5 × 10^8^	≈1.5 × 10^8^
	T_2_ (24 h)	0 × 10^0^	0 × 10^0^	0 × 10^0^	0 × 10^0^
	T_3_ (48 h)	0 × 10^0^	0 × 10^0^	0 × 10^0^	0 × 10^0^
*E. coli*	Control sample	1.5 × 10^8^	1.5 × 10^8^	1.5 × 10^8^	1.5 × 10^8^
	T_1_ (3 h)	≈1.5 × 10^8^	≈1.5 × 10^8^	≈1.5 × 10^8^	≈1.5 × 10^8^
	T_2_ (24 h)	5 × 10 ^0^	1 × 10 ^0^	1 × 10 ^0^	0 × 10^0^
	T_3_ (48 h)	0 × 10^0^	0 × 10^0^	0 × 10^0^	0 × 10^0^
*P. aeruginosa*	Control sample	1.5 × 10^8^	1.5 × 10^8^	1.5 × 10^8^	1.5 × 10^8^
	T_1_ (3 h)	≈1.5 × 10^8^	≈1.5 × 10^8^	≈1.5 × 10^8^	≈1.5 × 10^8^
	T_2_ (24 h)	0 × 10^0^	0 × 10^0^	0 × 10^0^	0 × 10^0^
	T_3_ (48 h)	0 × 10^0^	0 × 10^0^	0 × 10^0^	0 × 10^0^
*C. albicans*	Control sample	1.5 × 10^8^	1.5 × 10^8^	1.5 × 10^8^	1.5 × 10^8^
	T_1_ (3 h)	≈1.5 × 10^8^	≈1.5 × 10^8^	≈1.5 × 10^8^	≈1.5 × 10^8^
	T_2_ (24 h)	0 × 10^0^	0 × 10^0^	0 × 10^0^	0 × 10^0^
	T_3_ (48 h)	0 × 10^0^	0 × 10^0^	0 × 10^0^	0 × 10^0^

## Data Availability

Not applicable.

## Supplementary Materials

The following supporting information can be downloaded at: https://www.mdpi.com/article/10.3390/polym15040877/s1, Figure S1: Experimental TG/DTG curves of PSFEtP^+^ sample; Figure S2: Experimental TG/DTG curves of PSFQ sample [46].
