# Supplementary material for: Structure-Bioactivity Relationship of the Functionalized Polysulfone with Triethylphosphonium Pendant Groups: Perspective for Biomedical Applications
Source: Polymers (Basel). 2023 Feb 10;15(4):877. doi: 10.3390/polym15040877 (PMC9959649; doi:10.3390/polym15040877)
Supplement: Supplementary file 1 [file polymers-15-00877-s001.zip › polymers-2190703-supplementary.pdf]

Supplementary Materials

Structure-bioactivity relationship of the functionalized polysulfone with triethylphosphonium pendant groups: Perspective for biomedical applications

Adina Maria Dobos[1], Adriana Popa[2], Cristina Mihaela Rimbu[3] and Anca Filimon[1],*

[1] Department of Polycondensation and Thermally Stable Polymers, "Petru Poni" Institute of Macromolecular 6 Chemistry, 700487 Iasi, Romania

[2] Coriolan Dragulescu" Institute of Chemistry, Mihai Viteazul Blv., 300223 Timisoara, Romania

[3] Department of Public Health, Faculty of Veterinary Medicine, "Ion Ionescu de la Brad", University of Agricultural Sciences and Veterinary Medicine, 8, Mihail Sadoveanu Alley, 707027 Iasi, Romania

* Correspondence: afilimon@icmpp.ro

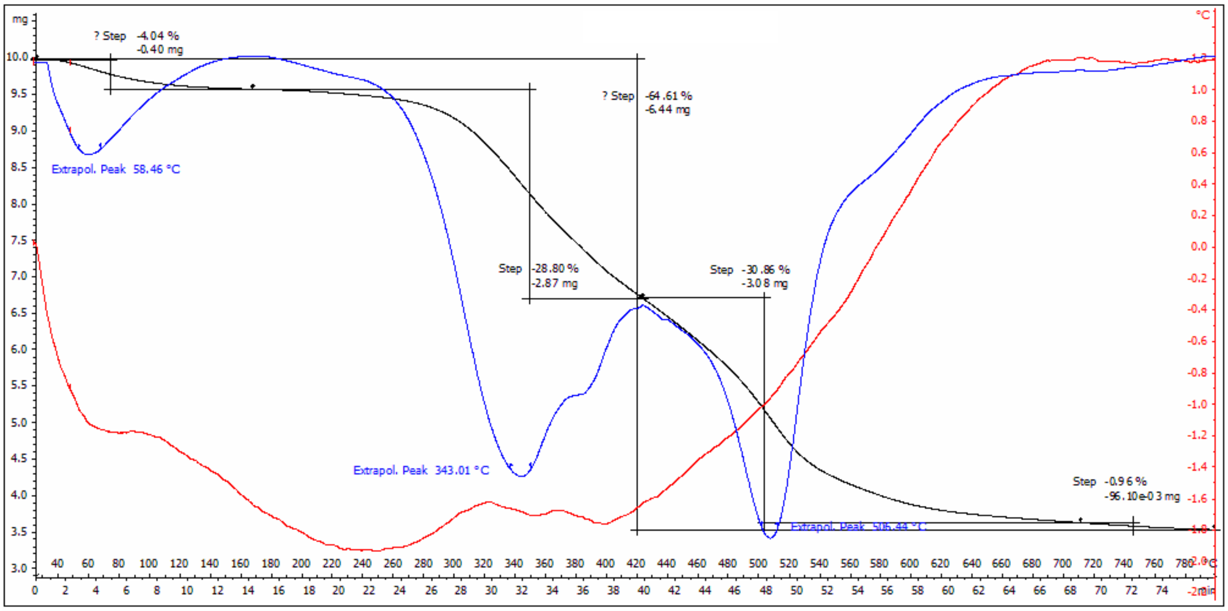

Figure S1. Experimental TG/DTG curves of PSFEtP+ sample.

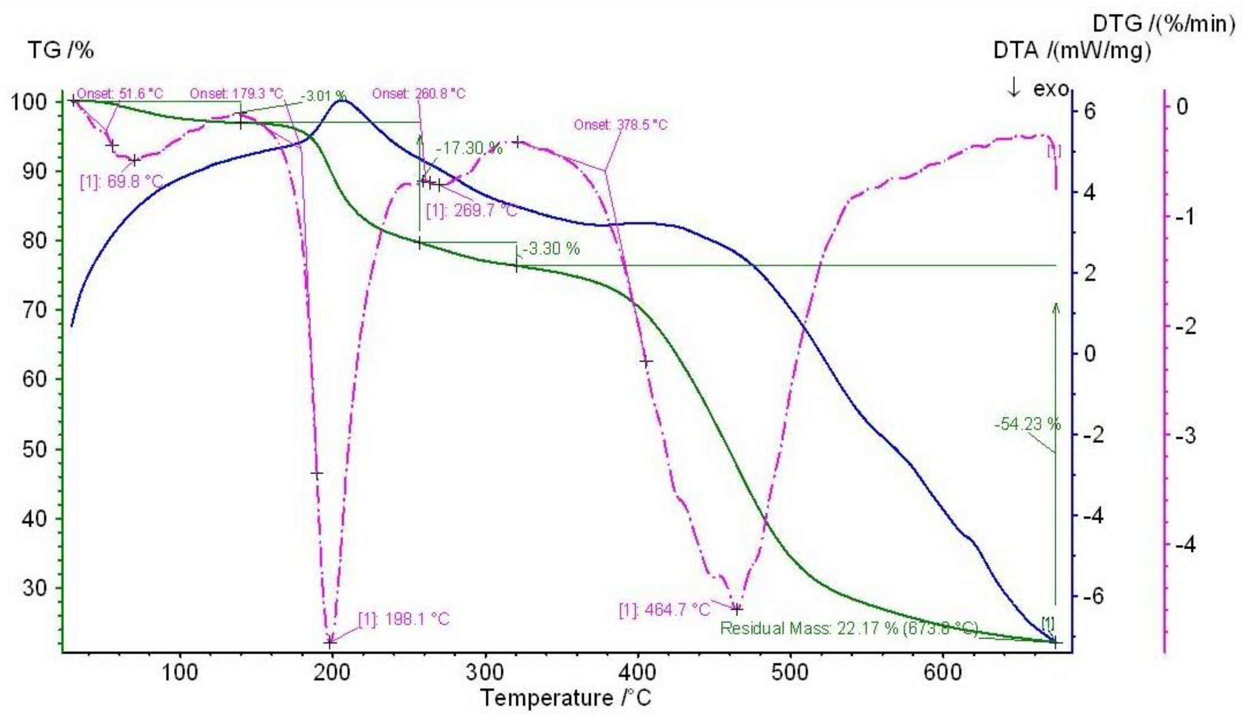

Figure S2. Experimental TG/DTG curves of PSFQ sample [46].
